# TET family proteins and 5-hydroxymethylcytosine in esophageal squamous cell carcinoma

**DOI:** 10.18632/oncotarget.4281

**Published:** 2015-06-08

**Authors:** Asuka Murata, Yoshifumi Baba, Takatsugu Ishimoto, Keisuke Miyake, Keisuke Kosumi, Kazuto Harada, Junji Kurashige, Shiro Iwagami, Yasuo Sakamoto, Yuji Miyamoto, Naoya Yoshida, Manabu Yamamoto, Shinya Oda, Masayuki Watanabe, Mitsuyoshi Nakao, Hideo Baba

**Affiliations:** ^1^ Department of Gastroenterological Surgery, Graduate School of Medical Science, Kumamoto University, Japan; ^2^ Department of Surgery, National Hospital Organization Kyushu Cancer Center, Japan; ^3^ Department of Cancer Biology, National Kyushu Cancer Center Clinical Research Institute, Japan; ^4^ Department of Gastroenterological Surgery, Cancer Institute Hospital, Japanese Foundation for Cancer Research, Japan; ^5^ Department of Medical Cell Biology, Institute of Molecular Embryology and Genetics, Kumamoto University, Japan

**Keywords:** epigenetics, markers, demethylation, TET

## Abstract

Mammalian DNA is epigenetically marked by 5′-cytosine methylation (5-methylcytosine [5-mC]). The Ten-eleven translocation (TET) enzymes (TET1, TET2, and TET3) are implicated in DNA demethylation, through dioxygenase activity that converts 5-mC to 5-hydroxymethylcytosine (5-hmC). Although decreased TET is reportedly associated with decreased 5-hmC levels in various cancers, functions of 5-hmC and TET expression in esophageal squamous cell carcinoma (ESCC) are unclear. We used ELISA and immunohistochemistry tests to analyze 5-hmC status in ESCC tissues, RT-qPCR to analyze *TET* family mRNA expression in normal and tumor tissues, and pyrosequencing to quantify LINE-1 (i.e., global DNA methylation) levels. ELISA and immunohistochemical testing showed 5-hmC levels were significantly lower in ESCC than in paired normal tissues (*P* < 0.0001). *TET2* expression was significantly lower in ESCCs than paired normal tissues (*P* < 0.0001), and significantly associated with 5-hmC levels in ESCCs (*P* = 0.003, *r* = 0.33). 5-hmC levels were also significantly associated with LINE-1 methylation level (*P* = 0.0002, *r* = 0.39). Patients with low 5-hmC levels had shorter overall survival than those with higher levels, although not significantly so (*P* = 0.084). In conclusion, 5-hmC expression was decreased in ESCC tissues, and was associated with *TET2* expression level. *TET2* reduction and subsequent 5-hmC loss might affect ESCC development.

## INTRODUCTION

Esophageal squamous cell carcinoma (ESCC), the most common esophageal cancer in East Asian countries, is a very aggressive malignancy that requires combined modality therapies [1]. However, the limited improvement provided by conventional therapies has prompted us to seek innovative strategies for treating ESCC, especially molecularly targeted treatments. Importantly, epigenetic changes, including alterations in DNA methylation, are reversible, and can thus be targets for therapy or chemoprevention [2–6].

DNA methylation—conversion of cytosine to 5-methylcytosine (5-mC)—is a primary epigenetic mechanism involved in imprinting, X-chromosome inactivation, and repression of endogenous retroviruses. In human cancers, DNA methylation alterations include global DNA hypomethylation and site-specific CpG island promoter hypermethylation, which leads to genomic instability or altered gene expression [7]. The ten–eleven translocation (TET) family proteins can convert 5-mC to 5-hydroxymethylcytosine (5-hmC), which is now widely recognized as the “sixth base” in the mammalian genome, following 5-mC, the “fifth base” [8–13]. 5-hmC is abundant in brain and embryonic stem cells, and is also distributed in many different human tissues [9, 10, 14, 15] Emerging evidence suggests that 5-hmC and TET family have unique functions in many processes such as gene control mechanisms and DNA methylation regulation, and affect many diseases, especially cancers. Loss of 5-hmC or TETs has been reported in several cancer types, such as myeloid leukemia, melanoma, and colorectal, breast, liver, and lung cancers [16–22]. However, no study has evaluated the status of 5-hmC and TETs expression in ESCC.

In this study, we examined the status of *TET*s and 5-hmC expression in matched ESCC and normal specimens, using ELIZA, immunohistochemistry (IHC) and RT-qPCR. We also quantified long interspersed nucleotide element-1 (LINE-1) methylation levels in these specimens, using pyrosequencing. As LINE-1 retrotransposons constitute a substantial portion of the human genome, LINE-1 methylation levels are regarded as surrogates for global DNA methylation [23]. Thus, we could comprehensively evaluate relationships among *TET*s, 5-hmC and LINE-1 methylation in ESCCs. Finally, we examined the prognostic value of 5-hmC and *TET* expression in patients with ESCC.

## RESULTS

### 5-hmC expression in ESCCs and matched normal mucosa

**Figure 1:**
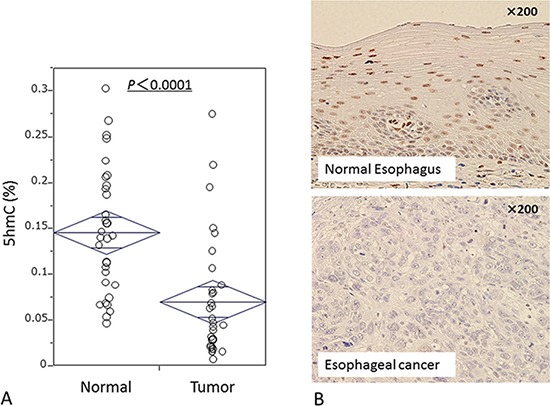
5-hmC expression in ESCC **A.** ELISA assay shows percentages of 5-hmC levels in 33 matched ESCC normal mucosa specimens. **B.** Immunohistochemical staining for 5-hmC in normal esophageal epithelium and ESCC.

To evaluate differences of 5-hmC status between esophageal cancer cells and normal epithelial cells, we first examined the 5-hmC content of nuclear DNA as % 5-hmC level by ELISA assay in 33 matched ESCC–normal esophageal mucosa pairs. Cancer tissues had significantly lower levels of 5-hmC (median: 0.046; mean: 0.070; standard deviation [SD]: 0.07) than matched normal mucosa (median: 0.141; mean: 0.146; SD: 0.07; *P* < 0.0001 by paired *t*-test) (Fig. 1A). Using 10 ESCC resected specimens, we confirmed by IHC that cancer cells had lower 5-hmC levels than did normal epithelial cells (Fig. 1B); nuclear cells of normal esophageal epithelial cells were immunoreactive for 5-hmC, whereas most cancer cells were not.

### TETs expression in ESCCs and matched normal mucosa

**Figure 2:**
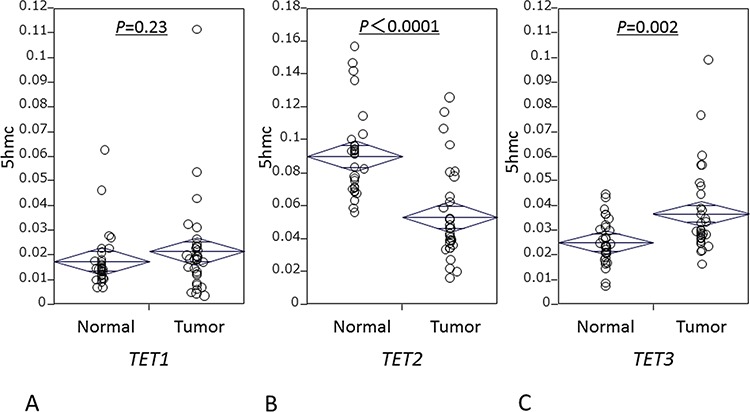
mRNA levels of TET family in 32 matched ESCC and matched normal mucosa specimens **A.** *TET1* expression. **B.** *TET2* expression. **C.** *TET3* expression.

We examined *TET* family mRNA levels in 32 ESCC specimens and matched normal esophageal mucosa utilizing frozen tissues in RT-qPCR assays. Levels of *TET1* expression were similar between cancer tissues (median: 0.019; mean: 0.022; SD: 0.02) and normal mucosa (median: 0.015; mean: 0.018; SD: 0.01; *P* = 0.23) (Fig. 2A). Notably, cancer tissues had significantly lower *TET2* levels (median: 0.045; mean: 0.053; SD: 0.03) than did normal mucosa (median: 0.087; mean: 0.090; SD 0.03; *P* < 0.0001, paired *t*-test; Fig. 2B); and significantly higher *TET3* levels (median: 0.030; mean: 0.037; SD: 0.032) than did normal mucosa (median 0.024; mean 0.026; SD 0.01; *P* = 0.0023; Fig. 2C).

### TETs expression and 5-hmC level in cancer tissues

**Figure 3:**
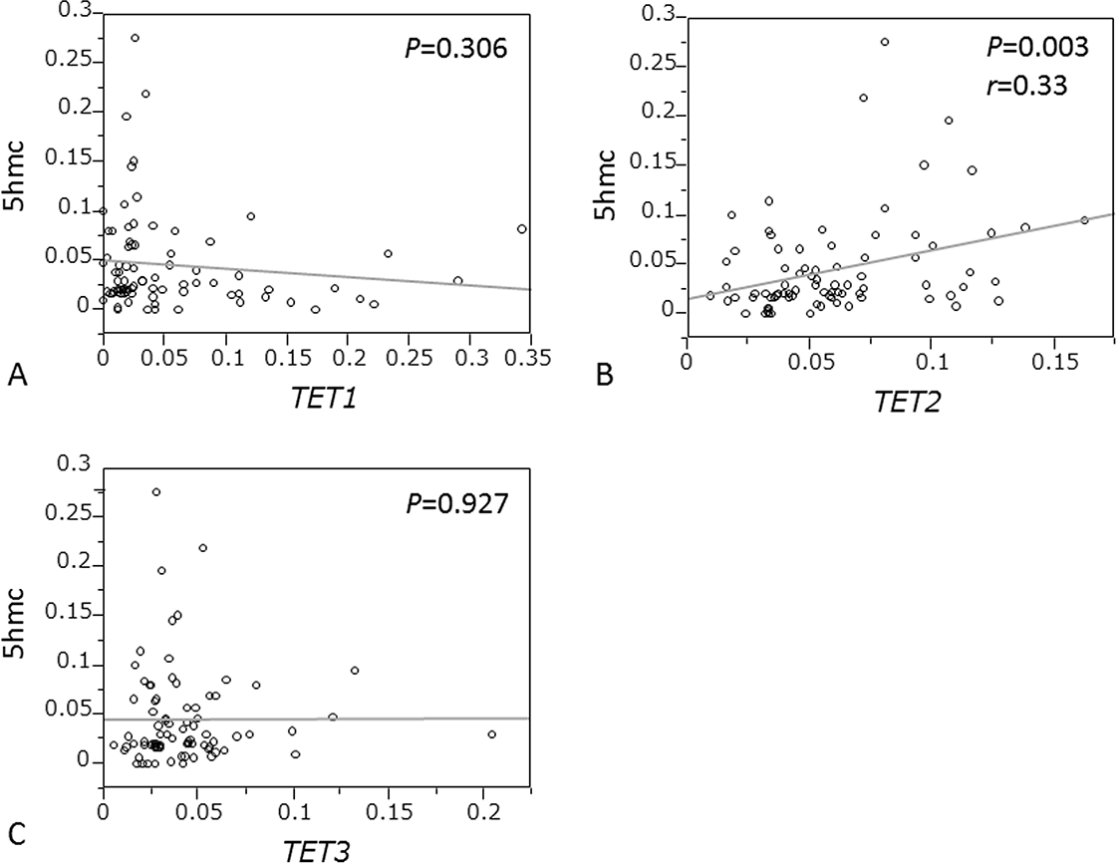
Correlation between 5-hmC expression and mRNA levels of TETs family in ESCC **A.** *TET1* expression. **B.** *TET2* expression. **C.** *TET3* expression.

We quantified the 5-hmC content of nuclear DNA as percentages of 5-hmC levels by ELISA in 95 ESCC specimens and obtained valid results in all cases, distribution of which over a 0–0.279 range was mean: 0.049; median: 0.029; SD, 0.050; and interquartile range, 0.016–0.068. We also evaluated expression levels of *TET1*, *2*, and *3* by RT-qPCR assay. We found that *TET2* expression was significantly associated with 5-hmC level (*P* = 0.003; *r* = 0.33 by paired *t*-test; Fig. 3B). However, *TET1* and *TET3* expression were not associated with 5-hmC level (*P* = 0.306 and *P* = 0.927, respectively; Fig. 3A, 3C).

### LINE-1 methylation level and 5-hmC level or TETs expression in cancer tissues

**Figure 4:**
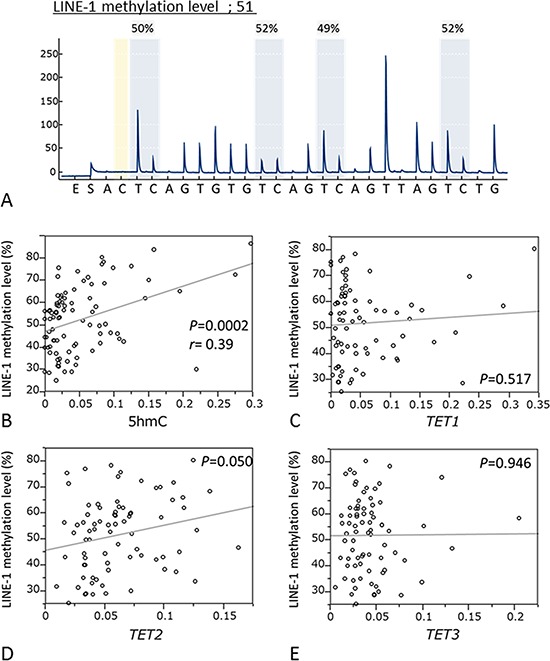
Correlation between the LINE-1 methylation levels and 5-hmC expression or mRNA levels of TETs family **A.** Pyrosequencing for LINE-1 methylation level. **B.** Relationship between LINE-1 methylation level and 5 hmC level. (*n* = 89, *P* = 0.0002, *r* = 0.39). **C.** Relationship between LINE-1 methylation level and TET1 expression. **D.** Relationship between LINE-1 methylation level and TET2 expression. **E.** Relationship between LINE-1 methylation level and TET3 expression.

LINE-1 methylation level is accepted as a surrogate marker for global DNA methylation level. Pyrosequencing was used to quantify LINE-1 methylation (Fig. 4A) to examine relationships between LINE-1 methylation and 5-hmC level or *TET* expression. We found 5-hmC levels were significantly associated with LINE-1 methylation (*P* = 0.0002; *r* = 0.39, paired *t*-test; Fig. 4B). However, *TET* mRNA expression was not associated with LINE-1 methylation (*TET1*: *P* = 0.517; *TET2*: *P* = 0.050; *TET3*: *P* = 0.946 respectively, paired *t*-test; Fig. 4C–4E).

### 5-hmC levels and clinical, epidemiological, and pathological variables

We assessed 5-hmC level against clinicopathological characteristics in 95 ESCCs, and found 5-hmC level was significantly associated with histologic grade (*P* = 0.017), but not with sex, age, year of operation, tobacco smoking, alcohol drinking, preoperative chemotherapy, tumor location, stage, or lymph node metastases (all *P* > 0.078; Table 1).

**Table 1:** Expression of 5-hmC in esophageal cancers, with clinical and tumor features

Clinical, epidemiologic or pathologic feature	Total N	5-hmC expression (%)		*P* value
		high	low	
All cases	95	48	47	
Mean age ± SD	68.3 ± 8.7	67.8 ± 8.1	68.8 ± 9.4	0.56
Sex				0.08
Male	86 (91%)	41 (85%)	45 (96%)	
Female	9 (9%)	7 (15%)	2 (4%)	
Tobacco use				0.53
Yes	81 (85%)	42 (88%)	39 (83%)	
No	14 (15%)	8 (12%)	8 (17%)	
Alcohol use				0.16
Yes	85 (89%)	45 (94%)	40 (85%)	
No	10 (11%)	3 (6%)	7 (15%)	
Year of diagnosis				0.23
2003 to 2007	14 (15%)	5 (10%)	9 (19%)	
2008 to 2012	81 (85%)	43 (90%)	38 (81%)	
Preoperative treatment				0.24
Present	38 (40%)	22 (46%)	16 (34%)	
Absent	57 (60%)	26 (54%)	32 (66%)	
Tumor location				0.14
High	9 (9%)	6 (13%)	3 (6%)	
Middle	47 (50%)	19 (40%)	28 (60%)	
Low	39 (41%)	23 (47%)	16 (34%)	
Stage				0.70
I (IA, IB)	12 (13%)	6 (13%)	6 (13%)	
II (IIA, IIB)	27 (29%)	13 (28%)	14 (30%)	
III (IIIA, IIIB, IIIC)	53 (57%)	27 (58%)	26 (57%)	
Lymph node metastasis				0.60
Positive	65 (68%)	34 (71%)	31 (66%)	
Negative	30 (32%)	14 (29%)	16 (34%)	
Histologic grade				0.017
G1	29 (31%)	19 (40%)	10 (21%)	
G2	46 (48%)	24 (50%)	22 (47%)	
G3–4	20 (21%)	5 (10%)	15 (32%)	

### TET2 expression and clinical, epidemiological, and pathological variables

As *TET2* expression was significantly associated with 5-hmC expression, *TET2* may be more important in ESCC development than *TET1* and *TET3*; we therefore evaluated relationships between *TET2* and clinicopathological characteristics. Distribution of *TET2* expression was mean: 0.060; median: 0.053; SD, 0.032; range: 0–0.16; and interquartile range: 0.034–0.075. *TET2* expression was not associated with sex, age, year of operation, tobacco smoking, alcohol drinking, preoperative chemotherapy, tumor location, stage, lymph node metastases, or histologic grade (all *P* > 0.215; supplemental table).

### 5-hmC and TET2 expression in ESCC and patient survival

**Figure 5:**
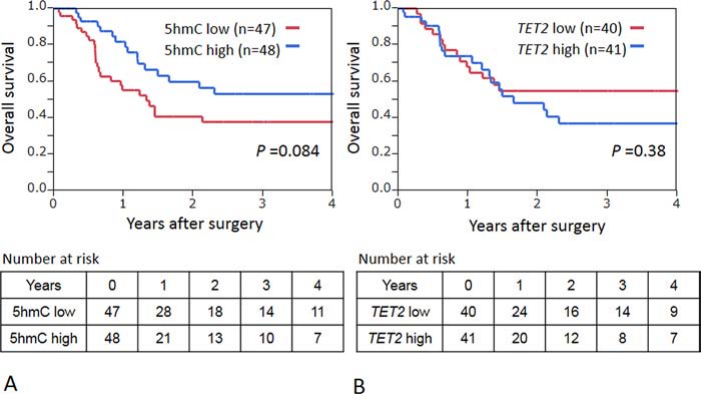
**A.** Kaplan–Meier curves for overall survival according to 5 hmC status in ESCCs. **B.** Kaplan–Meier curves for overall survival according to *TET2* stutus in ESCCs.

During an adequate follow-up period among the 95 patients, 46 patients died, including 26 deaths that were confirmed as attributable to ESCC. Median follow-up time for censored patients was 12.4 months. We divided patients into those who expressed high 5-hmC levels ( ≥ 0.029, *n* = 48) and the low 5-hmC group (0–0.028, *n* = 47). The low 5-hmC group experienced shorter overall survival than the high 5-hmC group, but not significantly so (log-rank *P* = 0.084; univariate hazard ratio = 1.68, 95% confidence interval (CI): 0.93–3.09, *P* = 0.086) (Fig. 5A). When patients were divided into those who expressed high *TET2* levels ( ≥ 0.053, *n* = 41) and low *TET2* (0.0059–0.0529, *n* = 40), Kaplan–Meier analysis showed the two groups to have similar overall mortality rates (log-rank *P* = 0.38; Fig. 5B). In addition, we found that *TET1* and *TET2* were not associated with patient prognosis (log-rank *P* = 0.39 for *TET1* and *P* = 0.95 for *TET3*).

## DISCUSSION

**Figure 6:**
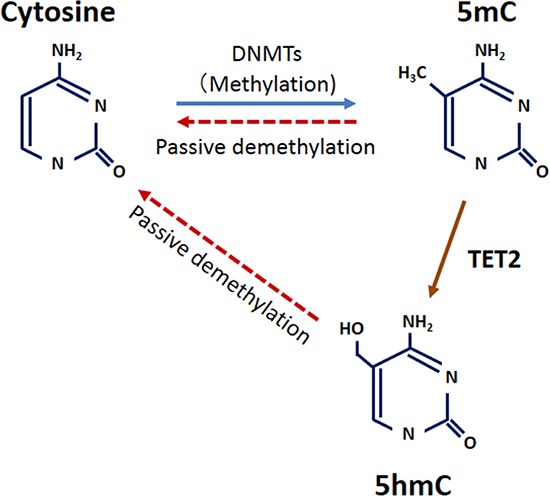
Model for the relationship between 5-hmC and TET2 in ESCC

This is the first study to examine expression levels of TET family and 5-hmC in esophageal cancer. Although DNA methylation and its mechanism (e.g., the DNA-methyltransferase [DNMT] family) are well-studied in human cancer, DNA demethylation is poorly understood. The TET family is apparently a key factor of DNA demethylation and can convert 5-mC to 5-hmC in mammalian cells. Our current study found that esophageal cancer tissues expressed lower levels of 5-hmC and *TET2* than did normal esophageal epithelium. Additionally, *TET2* expression was significantly associated with 5-hmC level in ESCCs, but *TET1* and *TET3* were not. Together, these results indicate that TET2 has a central function in converting 5-mC to 5-hmC in esophageal epithelial cells, and reduced TET2 causes decreased 5-hmC level in those cells, leading to ESCC development (Fig. 6).

The discovery of 5-hmC and TET enzymes as DNA modifications and modifiers (respectively) in mammalian genomes has raised many questions on the function of DNA demethylation in epigenetic regulation. Apparently, 5-hmC is not merely a marker, but has a mechanistic role in gene expression, [9, 31, 32] such as alteration of chromatin structure or contribution to the recruitment or exclusion of DNA-binding proteins. 5-hmC prevents DNMT-1-mediated methylation of target cytosines [33], thus leading to active demethylation [34]. Damage to 5-hmC in methyl-CpG sequences interferes with the ability of several methyl-CpG-binding proteins to bind to them, which may affect transcriptional activity of genes with significant levels of promotor-region 5-hmC [21, 35]. In view of the possibility that disordered 5-hmC plays a role in cancer development, better understanding of 5-hmC status is increasingly important in oncology. Reportedly, 5-hmC levels are reduced in several carcinomas including prostate, breast, colon and liver [36, 37]. The current study has shown similar phenomenon in ESCC tissues. Loss of 5-hmC is likely to be common in cancers, and to play an important role in carcinogenesis.

Decreased 5-hmC levels in malignant tumors are reportedly due to mutation or reduced expression of *TET1*/*2*/*3* genes [20, 22, 36, 38]. This study found that, of these three genes, only *TET2* expression was lower in ESCC than in normal epithelium, and was associated with 5-hmC level. These results imply that TET2 has a central function in converting 5-mC to 5-hmC in esophageal epithelial cells; and that its loss decreases 5-hmC in esophageal epithelial cells, subsequently leading to ESCC development. Recently, Lian et al has reported that 5-hmC is lost in melanoma and rebuilding the 5-hmC landscape in melanoma cells by reintroducing active *TET2* suppresses melanoma growth and increases tumor-free survival in animal models [22]. A study of hepatocellular carcinoma (HCC) has shown that only *TET1* expression was decreased in tumors relative to non-tumor tissues, indicating that *TET1* has an important function in converting 5-mC to 5-hmC in hepatocellular cells [36]. Different TET family members might participate in different types of cancers as tumor suppressors. Future studies are needed to confirm these findings, and to elucidate the mechanisms by which *TET2* loss leads to reduced 5-hmC level in esophageal epithelial cells and subsequent ESCC development.

The relationship between 5-hmC levels and patient prognosis has been reported in some cancer types, with lower 5-hmC significantly associated with shorter survival in intrahepatic cholangiocarcinoma, HCC, gastric cancer, and myelodysplastic syndromes [38–42]. We also found that low 5-hmC expression was loosely associated with poor prognosis in ESCC patients (although not significantly). The exact role and molecular mechanism of 5-hmC in human cancers are still unclear. The 5-hmC as an epigenetic modifier could be a marker for both early diagnosis and prognosis of cancer. Future studies are needed to confirm the association between 5-hmC and clinical outcome, and to examine potential mechanisms by which 5-hmC loss affects tumor behavior.

Interestingly, 5-hmC levels were significantly associated LINE-1 methylation levels. As LINE-1 represents a major repetitive element and occupies ∼17% of the human genome, LINE–1 methylation levels are regarded as a surrogate marker of global DNA methylation [23]. LINE-1 methylation has been shown to be vary widely in many human neoplasms; LINE-1 hypomethylation is strongly associated with a poor prognosis in several cancer types [27, 43, 44]. As 5-hmC is considered to be almost a sixth genomic base and possible intermediate in active DNA demethylation, our findings on the significant relationship between 5-hmC and LINE-1 methylation levels (i.e., 5-mC level in global DNA) indicate a delicate balance between 5-mC and 5-hmC in ESCC cells.

In summary, we found that 5-hmC expression was decreased in ESCC tissues, and was significantly associated with *TET2* expression level. Taken together, *TET2* reduction and subsequent 5-hmC loss might be crucial in ESCC development and progression.

## MATERIALS AND METHODS

### Study subjects

We initially enrolled 107 patients with ESCC who underwent curative resection at the Kumamoto University Hospital and the National Hospital Organization Kyushu Cancer Center between March 2003 and December 2012 in this study, of whom 12 patients were excluded owing to the unavailability of adequate fresh frozen tissue specimens. Finally, 67 patients from the Kumamoto University Hospital and 28 from the National Hospital Organization Kyushu Cancer Center were included in this study. Patients were observed at 1- to 3-month intervals until death or 31 December 2013, whichever came first. Overall survival was defined as the time between the date of surgery and date of death. Written informed consent was obtained from each subject, and the study procedures were approved by the institutional review board.

### DNA extraction and sodium bisulfite treatment

We extracted DNA samples from tumor and normal frozen surgical specimens, and isolated genomic DNA by standard procedures with phenol-chloroform and ethanol precipitation, using the protocol of Molecular Cloning (second edition), which was then modified with sodium bisulfite using an EpiTect Bisulfite Kit (Qiagen, Valencia, CA, USA).

### Enzyme-linked immunosorbent assay (ELISA) for 5-hmC quantification

The Quest 5-hmC™ DNA ELISA Kit (Zymo Research, Irvine, CA, USA) was used for colorimetric detection of 5-hmC, following the manufacturer's guidelines. Previous study utilized this ELISA Kit to detect the presence of 5-hmC in mammalian mitochondrial DNA [45]. Briefly, anti-5-hmC polyclonal antibody (1 μg/ml) and 100 μl of DNA binding solution were added to a 96-well plate and incubated at 37°C for 60 minutes; 100 ng of genomic DNA was added for 60 minutes, followed by 0.2 μg/ml of the Anti-DNA HRP antibody for 30 minutes. Absorbance was read at 450 nm. The amount of 5-hmC was calculated based on a standard curve generated using the kit controls.

### Pyrosequencing to measure LINE-1 methylation

Genomic DNA was extracted from the tumor and modified with sodium bisulfite using an EpiTect Bisulfite Kit (Qiagen). PCR and subsequent pyrosequencing for LINE-1 were performed as previously described by Ogino et al., using the PyroMark Kit (Qiagen) [24–27]. This assay amplifies a region of the LINE-1 element (position 305–331 in accession no. X58075), which includes four CpG sites. The amount of C relative to the sum of the amounts of C and T at each CpG site was calculated as a percentage (*i.e*., 0–100%). The average of the relative amounts of C in the four CpG sites was used as the overall LINE-1 methylation level in a given tumor. We validated our LINE-1 methylation pyrosequencing assay in the published literature [28].

### Quantitative real-time RT-PCR

Total RNA was extracted from tumor or normal frozen surgical specimens using ISOGENII (Nippon Gene, Tokyo, Japan). cDNA synthesis, and quantitative reverse transcription PCR (qRT-PCR) were carried out as previously described [29]. We used the SuperScript III Transcriptor First Strand cDNA Synthesis System for RT-PCR (Invitrogen, Carlsbad, CA, USA) to synthesize cDNA, according to manufacturers' instructions, and a LightCycler 480 II instrument (Roche Diagnostics, Mannheim, Germany) to perform qRT-PCR. We used the 2^−ΔΔCt^ method to determine differences in gene expression levels between specimens [30]. For qRT-PCR, primers were designed using the Universal Probe Library (Roche), following the manufacturer's recommendations. Expression of *TET* genes was normalized to that of *ARF*. Primer sequences and probes used for real-time PCR were: *TET1*-rt, 5′-TCTGTTGTTGTGCCTCTGGA-3′ (forward) and 5′-GCCTTTAAAACTTTGGGCTTC-3′ (reverse); *TET2*-rt, 5′-ACGCTTGGAAGCAGGAGAT-3′ (forward) and 5′-AAGGCTGCCCTCTAGTTGAA-3′ (reverse); *TET3*-rt, 5′-CGCCTCTATCCGGGAACT-3′ (forward) and 5′-TCCCCGTGTAGATGACCTTC-3′ (reverse); and *AR*F, 5′-TGCTGTCCTCCTGGTGTTC-3′ (forward) and 5′-CAGTTCCTGTGGCGTAGTGA-3′ (reverse).

### Immunohistochemical staining

Antibodies against 5-hmC (Active Motif, Carlsbad, CA, USA) were used as primary antibodies. The secondary antibody used was a ready-for-use anti-rabbit EnVision-Peroxidase system (Dako Japan Inc., Tokyo, Japan). Nuclear 5-hmC expression was recorded as negative, weak, or strong expression by an investigator (Y.B.) unaware of other data. 5-hmC-positive expression was defined as weak or strong expression in this study

### Statistical methods

For statistical analyses, we used JMP software (version 9; SAS Institute, Cary, NC). All *P* values were two-sided. To compare means, we performed *t*-tests that assumed unequal variances. For survival analysis, we used the Kaplan–Meier method to assess survival time distribution (log-rank test).

## SUPPLEMENTARY TABLE


