# Supplementary material for: TET family proteins and 5-hydroxymethylcytosine in esophageal squamous cell carcinoma
Source: Oncotarget. 2015 Jun 8;6(27):23372–82. doi: 10.18632/oncotarget.4281 (PMC4695124; doi:10.18632/oncotarget.4281)
Supplement: Supplementary file 1 [file oncotarget-06-23372-s001.pdf]

SUPPLEMENTARY TABLE

Supplementary Table S1. Expression of *Tet2* in esophageal cancers

Clinical, epidemiologic or pathologic feature	Total N	*Tet2* expression (%)		*P* value
		high	low	
All cases	81	41	40	
Mean age ± SD	68.1 ± 9.1	67.2 ± 8.5	69.0 ± 9.7	0.48
Sex				0.97
Male	75 (93%)	38 (93%)	37 (93%)	
Female	6 (7%)	3 (7%)	3 (7%)	
Tobacco use				0.78
Yes	70 (86%)	35 (86%)	35 (87%)	
No	11 (14%)	6 (14%)	3 (13%)	
Alcohol use				0.67
Yes	74 (91%)	38 (93%)	36 (90%)	
No	7 (9%)	3 (7%)	4 (10%)	
Year of diagnosis				0.96
2003 to 2007	14 (17%)	7 (17%)	7 (18%)	
2008 to 2012	67 (83%)	34 (83%)	33 (82%)	
Preoperative treatment				0.44
Present	33 (41%)	15 (37%)	18 (45%)	
Absent	48 (59%)	26 (63%)	22 (55%)	
Tumor location				0.38
High	8 (10%)	5 (12%)	3 (8%)	
Middle	41 (51%)	23 (56%)	18 (45%)	
Low	32 (39%)	13 (32%)	19 (47%)	
Stage				0.33
I (IA, IB)	12 (15%)	7 (17%)	5 (13%)	
II (IIA, IIB)	23 (28%)	14 (34%)	9 (22%)	
III (IIIA, IIIB, IIIC)	46 (57%)	20 (49%)	26 (65%)	
Lymph node metastasis				0.39
Positive	53 (65%)	25 (61%)	28 (70%)	
Negative	28 (35%)	16 (39%)	12 (30%)	
Histologic grade				0.21
G1	20 (28%)	12 (34%)	8 (22%)	
G2	35 (49%)	17 (49%)	18 (48%)	
G3–4	16 (22%)	5 (14%)	11 (30%)	
